# Hypertrophy of the Prostrate

**Published:** 1894-12-08

**Authors:** 


### Hypertrophy of the Prostate.

Disorders of the act of micturition are amongst the most important and the most interesting maladies met with in the male subject. The act of micturition may be briefly stated as consisting of a relaxation of the sphincter, and a contraction of the detrusor, which may be effected voluntarily, but are usually brought about by the stimulus of distention of the bladder. As might be supposed, this delicate mechanism may be very easily deranged, either from the nervous or the purely mechanical side. In the one case we have derangement of the brain, or spinal cord, or of the sensory surfaces, such as the skin, the bladder, the prostatic urethra, or the rectum; in the other the cause lies in some impediment to the flow of urine, either from calculus, tumour, stricture, or enlargement of the prostate. Certain of these conditions are apt to occur at certain periods of life, and even the normal act of micturition varies according to the age of the patient. Thus, in the child micturition is very easily started and cannot be stopped at will; the adult is able to stop in the middle; the old man has difficulty in starting the act.

Thompson has laid down some important generalisations of great diagnostic value, in regard to impeded or frequent micturition. Such conditions, in an otherwise healthy man of from eighteen to twenty-five years of age, are probably due to some inflammatory action in the bladder or urethra; in a man of from twenty-five to fifty-five years old they may be due to the same cause, but rather point to stricture; at or after fifty-six years of age they are often due to hypertrophy of the prostate.

The form of hypertrophy to which the prostate is subject is an affection peculiar to this organ, and is in no sense a new formation. The only other condition that is at all analogous is fibro-myoma of the uterus. Whilst hypertrophy of the prostate is essentially a senile complaint, it is by no means a necessary accompaniment of grey hair, or of arcus senilis, as was at one time supposed, and its cause is still a disputed point. Amongst prominent symptoms we may mention diminished force of the stream during micturition, inability on the part of the patient to alter this at will, delay before the act begins, and frequency of micturition, especially at night. There may be involuntary passage of urine, a condition almost always associated with retention rather than incontinence. This symptom has been erroneously attributed to "incontinence" and even "paralysis," but the "dribbling" which occurs from hypertrophy of the prostate is in reality an overflow from a bladder distended, in consequence of some mechanical impediment to the outflow from it, and when the distension is for the time relieved, there remains behind a certain amount of residual urine. The patient will say he is "making plenty of water"; and the surgeon may be led to suppose that the patient cannot retain his urine. Medicine is powerless to diminish the size of the prostate, but may be of value in relieving any local congestion. The cause of the trouble is mechanical, and it is to mechanical means that we must turn in the treatment of these cases, and thus, as a rule, the patient must be instructed how to enter upon "catheter life."

But it is by no means uncommon for a patient, who has long been the subject of hypertrophied prostate, to be attacked, quite suddenly, with complete retention. The condition is one of "extreme distress, often indeed of imminent danger." It is not surprising, therefore, to find that, in order to avoid so serious a contingency, the surgeon has attempted a "radical cure." A good many operations have now been performed for this condition. Whitehead makes a permanent perineal opening, after perineal urethrotomy. McGuire is in favour of a permanent supra-pubic urethra. Others have gone still further, and by attacking the gland itself have succeeded in removing the cause of the obstruction by what is known as McGill's operation of supra-pubic prostatectomy.

We have referred to the analogy between hypertrophy of the prostate and myoma uteri. This pathological analogy has suggested analogous treatment, and has given rise to two other methods of procedure, having for their object the reduction in the size of the gland by a diminution of its blood supply, and an alteration of its nervous connections. From the good results following ligation of the uterine arteries for myoma uteri, of the thyroid arteries in cases of goitre, and of the nutrient arteries in fibroid tumours of the breast, it occurred to Bier, of Kiel, that the size of the prostate might be reduced in the same way by ligature of the internal iliac arteries, preferably simultaneously. The chief fear that there might be danger in ligaturing such large vessels in old people in consequence of atheroma, and liability to secondary hæmorrhage, is not borne out by facts, although there are some who hold that hypertrophy of the prostate is due to a local and general arterio-sclerosis. The results of this method have so far proved very promising, and, according to Meyer, it seems safe to predict a progressive and permanently lasting atrophy of the prostate from ligature of the internal iliacs in cases where it is hypertrophied.

Again, from the fact that fibro-myoma of the uterus has been known to disappear after oöpherectomy, it seemed reasonable to expect that hypertrophy of the prostate might subside after removal of the testes. It has, moreover, been observed by Griffiths and others that the prostate has undergone marked shrinkage, not only in dogs and in cats, but also in man after castration, and this observation has been confirmed by White in his experiments on dogs. Castration has, therefore, become a recognised method of treatment for hypertrophied prostate, and, according to Fremont Smith, its freedom from danger recommends it in a class of cases most unfitted by the infirmity of age, and a harassing disease, to undergo a surgical strain of any moment.

